# Study on Consumer Preference for Traceable Pork With Animal Welfare Attribute

**DOI:** 10.3389/fpsyg.2021.675554

**Published:** 2021-07-01

**Authors:** Mo Chen, Enhua Hu, Lin Lin Kuen, Linhai Wu

**Affiliations:** ^1^School of Economics and Management, Nanjing University of Aeronautics and Astronautics, Nanjing, China; ^2^Department of Business Administration, Cheng Shiu University, Kaohsiung, Taiwan; ^3^Research Institute for Food Safety Risk Management, School of Business, Jiangnan University, Wuxi, China

**Keywords:** traceable pork, animal welfare, origin, food safety, bayesian inference

## Abstract

We determined consumer preferences for traceable pork attributes in 328 consumers in Wuxi City, Jiangsu Province, China, based on a traceable pork attribute system composed of traceability, animal welfare, place of origin, and price attributes. Preference was studied using a Choice Experiment and Bayesian inference analysis. Results showed that the marginal utility of health welfare was lower than that of high-level traceability information and similar to that of place of origin but was higher than that of middle-level traceability information. A complementary relationship existed between dietary animal welfare and high-level traceability information and between health welfare and non-indigenous production. A substitution relationship existed between health welfare and indigenous production and between environmental animal welfare and non-indigenous production. The marginal utilities of health welfare and dietary welfare were higher than those of all price levels, and consumers accept a higher price as a result of increased production costs due to the inclusion of animal welfare information. Due to the harsh realities of COVID-19, China has recently approved the animal welfare attribute to be integrated into traceability market systems of new animal-derived food. The government should encourage manufacturers to produce diverse traceable animal-derived food not only to protect animal welfare and promote the construction of an ecological civilization, but also to develop new animal-derived food markets to satisfy different levels of consumer demand.

## Introduction

Since March 2020, COVID-19 has rapidly spread worldwide, resulting in incalculable losses to humans. The COVID-19 pandemic has also triggered global reflection on animal welfare protection, including the consumption of wild animals as food, and more deeply, the impact of animal health on human health. For example, according to the Ministry of Agriculture and Rural Affairs of the People's Republic of China, since the initial outbreak of African Swine Fever (ASF) in August 2018 to January 2020, China has reported 162 outbreaks and culled nearly 1.2 million infected pigs, resulting in huge economic losses to the pork industry. However, although the government has taken strict action against ASF prevention, ASF-infected pork is still present in the market, thus disturbing normal market order and threatening pork quality and safety, and consequently human health and safety. For example, the Public Security Bureau of Rui'an, Wenzhou, Zhejiang, as disclosed in the Top Ten Food Crime Cases in the First Half of 2019 issued by the Zhejiang Provincial Public Security Department, investigated and uncovered the production and sale of food that did not meet safety standards, leading to the seizure of nearly 7 tons of ASF-infected pork and an illegal profit of more than two million yuan.^1^ Although ASF is not a zoonotic disease, nor is it considered infectious or harmful to humans, ASF-infected pork in the market has once again aroused widespread public concern about pork safety issues. Beltrán-Alcrudo et al. (2017) showed that the emergence and spread of ASF is largely caused by non-standard behavior of stakeholders in the pork supply chain system, as well as poor environment and management in pig farming, so that the physical well-being of pigs cannot be guaranteed. For example, swill, domestic waste, and other pollutants are often used illegally to feed pigs and the farming environment is often unhygienic, resulting in disease and cross-infection due to bacterial proliferation.

In 1976, the American researcher Hughes introduced the concept of animal welfare for the first time. He defined farm animal welfare as a state of complete mental and physical health, where the animal is in harmony with its environment, advocating that humans should consider animal welfare while using animals humanely (Ren, 2006). Gavinelli et al. (2007) pointed out that animal-derived food safety will be impacted if basic animal welfare is neglected or cannot be guaranteed, which poses long-term potential threats to human health through the food chain. Iannetti et al. (2019) showed that poor animal welfare can lead to an increase in the probability of animal diseases and their potential transmission to humans. Among infectious diseases that threaten human health, there are more than 200 infectious zoonoses. Grace et al. (2012) defined zoonosis as an infectious disease transmissible among animals and humans. Grace et al. (2011) also reported that approximately 20% of human diseases and deaths in less developed areas are caused by zoonoses. As of 2019, an average of 12 million people worldwide die from zoonoses each year (Li et al., 2019). Iannetti et al. (2019) found that zoonoses have accounted for more than 70% of emerging infectious diseases in the past 30 years. The European Food Safety Authority (ESFA) (2019) emphasized that the susceptibility of animal-derived food to disease, including zoonoses, will increase if animal welfare cannot be guaranteed. Therefore, many European and American countries have established clear regulations regarding the protection of animal welfare in the process of farming, slaughter, and transportation from the perspective of food safety and social ethics. At present, the international community has adopted the basic definition given by the British Farm Animal Welfare Council (FAWC). The Farm Animal Welfare Council: Five Freedoms (2009) holds that animals should be entitled to five freedoms: i.e., freedom from hunger and thirst by ready access to fresh water and a diet to maintain full health and vigor (i.e., dietary welfare); freedom from discomfort by providing an appropriate environment including shelter and a comfortable resting area (i.e., environmental welfare); freedom from pain, injury, and disease by prevention or rapid diagnosis and treatment (i.e., health welfare); freedom to perform normal behavior by providing sufficient space, proper facilities, and adequate company of the animal's own kind (i.e., behavioral welfare); and freedom from fear and distress by ensuring conditions and treatments that avoid mental suffering (i.e., mental welfare). The World Health Organization has further stated that if animals are healthy, comfortable, fed, safe, able to express their nature freely, and free from pain, fear, and pressure, then the basic requirements of animal welfare have been met (Office International Des Epizooties (OIE), 2015). However, there is almost no legislation safeguarding animal welfare in China, and consumers know very little about animal welfare, which is the main cause of livestock meat quality and safety issues in China (Wang and Gu, 2016).

Many studies have shown that food traceability systems with both ex ante quality assurance (also known as ex ante warning) and ex post traceability can help eliminate information asymmetry between producers and consumers, prevent food safety problems, and reduce the impact of food safety incidents through traceability (Opara, 2003; Kher et al., 2013; Hou et al., 2019). Therefore, if an animal welfare information attribute with the function of ex ante quality assurance is added to pork traceability systems, it will help guide farmers to safeguard pig welfare in the farming process and prevent swine fever and other safety incidents, thereby achieving ex ante quality assurance (Alfnes et al., 2018). Furthermore, once an incident similar to ASF-infected pork entering the market occurs, pork that fails the required standard can be recalled in a timely manner, and those responsible can be held accountable, thereby achieving ex post traceability. However, adding an animal welfare information attribute to a pork traceability system will inevitably increase the production costs of pork (Weerd and Day, 2009), which will certainly be reflected in market prices. Therefore, whether consumers are willing to pay a certain premium for traceable pork with an animal welfare information attribute will affect the willingness of producers to produce traceable pork with this attribute. As illustrated by the literature review below, consumers in different countries are willing to pay a certain premium for animal welfare at different levels. However, similar research remains scarce in China. Based on the actual market situation in China, we carried out a case study on consumers in Wuxi City, Jiangsu Province, which incorporated an animal welfare information attribute into a traceable pork attribute system as ex ante quality assurance information. Thus, we built a traceable pork attribute system that integrated traceability information, animal welfare, place of origin, and price attributes. In addition, a Choice Experiment was employed to collect experimental data and Bayesian inference was used for data analysis to study the perceptions and preferences of consumers for traceable pork with the animal welfare information attribute. This provides a theoretical basis for the construction of a pork traceability system that includes an animal welfare attribute in China. Ultimately, it is hoped that this will enhance pork traceability systems to improve pork safety and ensure food safety and consumer health in China.

## Literature Review

As the food supply chain becomes increasingly complex, insufficient consumer knowledge on food safety information attributes can have a negative impact on the feedback loop of the supply chain (Reardon and Timmer, 2012). Regattieri et al. (2007) showed that food traceability systems provide consumers with quality and safety information and are an essential tool to prevent food safety risks. Based on the theory of consumer demand put forward by Lancaster (1996), the value of a commodity is created by the combination of its attributes. Therefore, the utility of consumers' consumption of food can be regarded as coming from a combination of attributes, including traceability. Food attributes can be divided into three categories, i.e., search, experience, and trust attributes (Wu et al., 2015a). Hobbs (2004) stated that a food traceability system is designed to provide two basic functions, i.e., ex ante quality assurance and ex post traceability. The main function of ex post traceability is that sub-standard food can be effectively recalled through the traceability system (Ubilava and Foster, 2009). Ex ante quality assurance presents consumers with trust attributes, such as food quality and safety, place of origin, and animal welfare, in the form of a label. This transforms the trust attribute of food safety into a search attribute, thereby reducing the time to search for desirable traceable foods (Golan et al., 2003) and playing a pre-warning role (Wu et al., 2015a). Various studies have shown that ex ante quality assurance plays a far greater role than ex post traceability in eliminating information asymmetry (Hobbs, 2004; Loebnitz and Loose, 2015).

Considerable research has been conducted on consumer preference for food traceability information with the function of ex post traceability. Loureiro and Umberger, 2007 adopted a Choice Experiment to study the preference of American consumers for safety attributes of meat products and found that such consumers pay more attention to products with food safety labels issued by the USDA, which prove that the meat is fresh and traceable, compared to products without safety labels. Abidoye et al. (2011) adopted the Choice-Based Conjoint approach to study American consumer preference for quality attributes of beef and concluded that such consumers are most concerned with and willing to pay a certain premium for traceability information. Bai et al. (2013) used the same method to study Chinese consumer preference for traceable milk and found that urban consumers prefer milk with traceability information. Using a Real Choice Experiment (i.e., real exchange of goods and money), Wu et al. (2015b) found that Chinese consumers have significantly heterogeneous preferences for traceable pork, and they are willing to pay a certain premium for traceability information on slaughter, processing, distribution, and sales. Furthermore, based on a Choice Experiment, Yin et al. (2017) showed that Chinese consumers prefer the traceability information attribute in the purchase of baby milk, for which they are willing to pay a certain premium. Wu et al. (2018) also studied the willingness of Chinese consumers to pay for pork with different levels of traceability information, confirming that consumers have the highest willingness to pay for complete traceability information that covers farming, slaughter, processing, distribution, and sales. Based on an Experimental Auction, Nguyen et al. (2018) studied Vietnamese consumers' willingness to pay for rice and found that the premium paid by consumers for rice gradually increased from 9% to 33% when the certified sustainably produced rice contained traceability information.

Scholars have also studied consumer preferences for place of origin and animal welfare attributes with the function of ex ante quality assurance. In terms of the place of origin attribute, Chang et al. (2013) found that American consumers prefer indigenous ground beef to ground beef from different origins. Lim et al. (2014) reported that American consumers have greater trust in the safety of domestic beef than imported beef and have a higher willingness to pay for beef with a domestic production label. In terms of the animal welfare attribute, Yuta et al. (2018) showed that nearly 90% of Japanese consumers are willing to pay a certain premium for beef with an animal welfare label. Markova-Nenova and Wätzold (2018) found that German consumers have a higher willingness to pay for milk with an animal welfare attribute. Lemos Teixeira et al. (2018) found that consumers in Brazil and Chile prefer eggs provided by farms that can guarantee animal welfare in terms of favorable diet, living conditions, and health. Merlino et al. (2018) confirmed that Italian consumers strongly consider animal welfare factors, second only to price factors, when purchasing beef. Spain et al. (2018) reported that 78% of American consumers believe that a fair and objective third party is required to ensure the reliability of animal welfare certification, and they are willing to pay a 32% premium for eggs under reliable animal welfare certification. Lai et al. (2018) also found that Chinese consumers in economically prosperous cities prefer pork with animal welfare labels, and are willing to pay a premium for them, but at a lower level than for food safety attributes.

The penetration rate of traceable pork in China is still low and the different types of traceable pork studied in this paper do not actually exist in the market (i.e., a virtual traceable pork profile). This makes it difficult to obtain actual purchase data on consumers' market behavior. Thus, consumers must be asked directly about their stated preferences and willingness to pay for traceable pork. Several basic methods can be applied to study stated preference, including Choice Experiment, Contingent Valuation, and Conjoint Analysis. According to Louviere et al. (2010), a Choice Experiment can be conducted with random utility theory as a starting point and have a mature practical basis, and thus has become a key tool to study consumer preferences.

In summary, various studies have investigated consumer preferences and have consistently reported that consumers generally pay attention to traceability, animal welfare, and place of origin. However, most previous studies on animal welfare have been conducted in developed countries, with studies on Chinese consumers and the incorporation of animal welfare into traceability systems with ex ante quality assurance remaining relatively scarce. Moreover, most earlier studies have used latent class modeling analysis tools to highlight group differences in consumers, while neglecting differences in consumers' individual preferences for different attributes. In the present study, we investigated the perceptions and preferences of consumers in Wuxi, Jiangsu Province, China, for an animal welfare attribute of traceable pork in a system composed of traceability, animal welfare, place of origin, and price attributes using a Choice Experiment and Bayesian inference analysis. Moreover, animal welfare and place of origin were classified as ex ante quality assurance attributes and traceability information was classified as an ex post traceability attribute according to Hobbs (2004). The results of this study should provide guidelines for the development and popularization of the traceable pork system that incorporates animal welfare in China.

## Experimental Design and Investigation

Based on the assumption that traceable pork can be regarded as a combination of traceability information, animal welfare, place of origin, and price attributes according to the utility theory proposed by Lancaster (1996), the experiment was designed and conducted as follows.

### Traceable Pork Attribute Setting

As the most frequently consumed meat in China, pork also has the most quality and safety incidents (Wu et al., 2015c). In 2018, pork output and consumption in mainland China amounted to 54.04 and 55.398 million tons, respectively, accounting for 47.82% and 49.25% of the world's total pork output and consumption, respectively. Moreover, traceable pork is one of the earliest traceable food types in the Chinese market. Therefore, we investigated traceable pork as a case study in this paper. Given the different consumer preferences for different parts of traceable pork of the same variety, pork hindquarters, a pork part widely consumed in China (Wang et al., 2011), were selected as the experimental product to eliminate the possible impact of non-intrinsic factors on research conclusions. For simplicity, traceable pork hindquarters are referred to as traceable pork hereinafter. The specific attribute settings are shown in Table 1.

**Table 1:** Attributes and levels of traceable pork.

**Category**	**Attribute**	**Level and definition**
*ex post* traceability	1. Traceability information	1. Information about farming, slaughter, and sales (HITRACE) 2. Information about farming and slaughter (METRACE) 3. Information about farming (LOTRACE) 4. No traceability information (NOTRACE)
*ex ante* quality assurance	2. Animal welfare	1. Dietary welfare (PHYSICAL) 2. Environmental welfare (ENVIR) 3. Health welfare (HEALTH) 4. No animal welfare (NOWELFARE)
	3. Origin	1. Indigenous (LOCORIGIN) 2. Non-indigenous (OTHORIGIN) 3. NOORIGIN
	4. Price	1.14 yuan/500 g (PRICE1) 2. 15.4 yuan/500 g (PRICE2) 3. 16.8 yuan/500 g (PRICE3) 4. 18.2 yuan/500 g (PRICE4)

**Figure 1:**
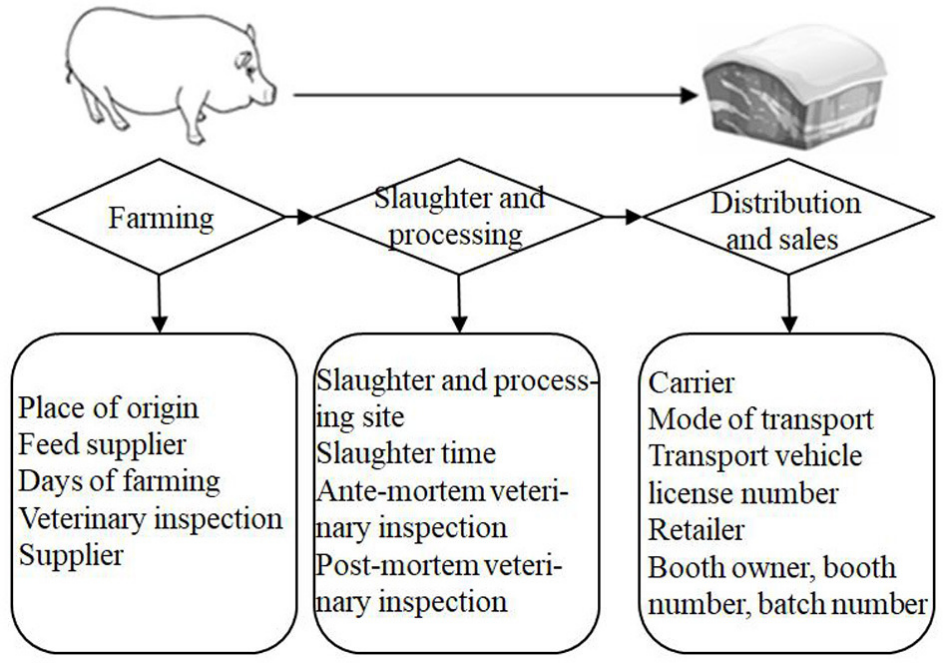
Schematic of traceability information in each link of the traceable pork supply chain.

First, the traceability information attributes and levels were set as follows. Wu et al. (2015c) found that food safety issues in China, including those of pork, are predominantly caused by human factors, such as improper behavior, failure to implement or strictly implement existing technical specifications and standard systems for food, and other violations related to production and business. From the perspective of the whole pork supply chain in China, and based on Wang et al. (2017), feed suppliers, farmers, butchers, dealers, and retailers are important stakeholders in the pork supply chain system, and their behaviors directly affect pork quality and safety. Due to information asymmetry on pork safety attributes, it is difficult for consumers to have full access to all relevant information, which thus leads to market failure (Wu et al., 2017). Therefore, information on traceable pork was set to cover three key links, i.e., farming, slaughter and processing, and distribution and sales. Moreover, the traceability information was displayed graphically to facilitate consumer understanding (Figure 1). Specifically, the traceability information attributes were designed with reference to the four levels shown in Table 1.

Second, animal welfare was introduced as an ex ante quality assurance attribute. Inspired by the Farm Animal Welfare Council: Five Freedoms (2009), three forms of animal welfare, i.e., dietary, environmental, and health welfare, were selected as attribute levels. In the design of the study questionnaire, the specific connotations of the three kinds of animal welfare were described clearly, and the following items were established accordingly, i.e., “how important is providing pigs with ready access to a satisfactory diet”; “how important is providing pigs with a well-ventilated pigsty that allows comfortable rest and activity”; “how important is providing pigs with access to immediate treatment when sick”, to ensure that surveyed consumers (hereinafter referred to as participants) had a direct perception of animal welfare. Five response options were included, i.e., “very unimportant”, “unimportant”, “neither important nor unimportant”, “important”, and “very important”.

**Figure 2:**
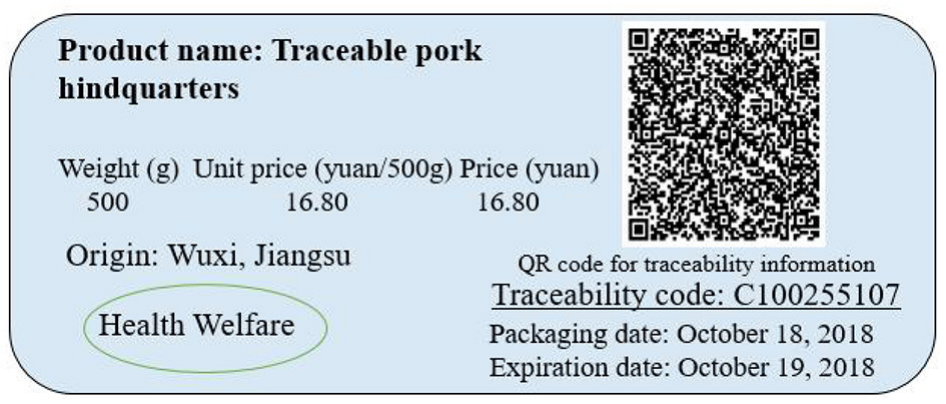
Experimental sample label of traceable pork hindquarters.

Third, the place of origin attribute was introduced into the traceable pork attribute system and separately listed in the label as an ex ante quality assurance attribute. Strictly speaking, the farming information in the traceability attribute established in this paper contains information on place of origin. However, Zhong and Wu (2018) showed that the scale of pig farming in China is still small at present and decentralized small-scale farming prevails, and thus the existing traceability system is unable to trace every small-scale pig farmer in the farming link. Although it is difficult for the place of origin information, as an independent attribute, to trace specific pig farmers, it can reflect the characteristics of the region where small-scale pig farmers are located. Moreover, it is necessary to include place of origin as an independent attribute of traceable pork in the context of China. Specifically, food safety in China is often associated with region and is closely related to regional natural environments (e.g., soil, water, and atmosphere) and social integrity.^2^ For example, heavy metal pollution in rice exhibits regional distribution in China (Wu et al., 2014). According to the Chinese definition, the place of origin of products includes regional space and geographical indication. Therefore, according to Wu et al. (2017), place of origin was considered an independent attribute of traceable pork in this study^3^ to provide consumers with producer-identifying information and thus with ex ante warning (Lim et al., 2014). In this paper, “LOCORIGIN” is defined as pork produced in Wuxi, where the experiment was conducted; “OTHORIGIN” is defined as pork produced in Lu'an City, Anhui Province, which is commonly available in the Wuxi market according to our local survey; and “NOORIGIN” is defined as pork without a specified place of origin. The place of origin attribute was labeled separately, as shown in Figure 2.

Fourth, properly setting the price of traceable pork profiles with different attributes and attribute levels is critical for Choice Experiments as all traceable pork products set in this paper do not exist in the actual market. As the site of the experiment and the selection of participants were from downtown Wuxi, consistent with those in Wu et al. (2018), and the time interval between these experiments was short, the price of pork set by Wu et al. (2018) was adopted here (see Table 2 for specific price levels).

**Table 2:** Demographics of participants.

**Demographic**	**Category**	**Sample size (person)**	**Proportion (%)**
Gender	Male	155	47.26
	Female	173	52.74
Age	18–25 years old	155	47.26
	26–35 years old	89	27.13
	36–45 years old	29	8.84
	46–55 years old	33	10.06
	56–65 years old	18	5.49
	66–72 years old	4	1.22
Marital status	Married	144	43.90
	Unmarried	184	56.10
Education background	Junior high school and below	39	11.89
	Senior high school (including vocational high school)	47	14.33
	Junior college (including higher vocational college)	64	19.51
	Bachelor's degree	143	43.60
	Master's degree or above	35	10.67
Children under 18 years old in the family	N	219	66.77
	Y	109	33.23
Pregnant or breast-feeding women in the family	N	308	93.90
	Y	20	6.10
Health condition	Very good	126	38.41
	Good	153	46.65
	Moderate	48	14.63
	Poor	1	0.31
	Very poor	0	0
Personal annual income	36 000 yuan and below	128	39.02
	36 000–50 000 yuan	64	19.51
	50 000–80 000 yuan	51	15.55
	80 000–100 000 yuan	38	11.59
	Above 100 000 yuan	47	14.33
Annual household income	50 000 yuan and below	33	10.06
	50 000–80 000 yuan	56	17.08
	80 000–100 000 yuan	62	18.90
	100 000–150 000 yuan	53	16.16
	Above 150 000 yuan	124	37.80
Number of family members	1	3	0.91
	2	30	9.15
	3	144	43.90
	4	79	24.09
	5 and above	72	21.95
Household pork consumption per week	500 g and below	43	13.11
	500–1 000 g	121	36.89
	1 000–1 500 g	92	28.05
	1 500–2 000 g	30	9.15
	Above 2 500 g	42	12.80

### Experimental Design

**Figure 3:**
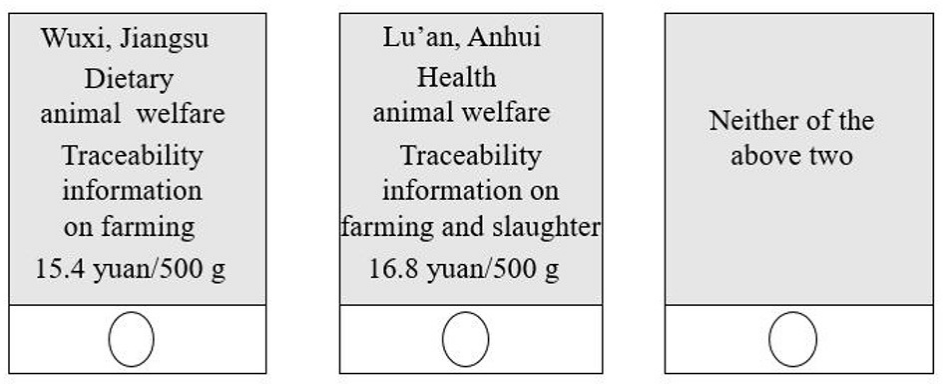
Single task sample of choice experiment.^4^

Each traceable pork attribute shown in Table 1 has a different number of levels. Therefore, the Choice Experiment followed a full factorial design (Louviere et al., 2000). The traceable pork attributes and attribute levels in Table 1 resulted in 4 × 3 × 3 × 4 = 144 virtual traceable pork profiles. Generally speaking, choice tasks that take more than 30 min will exhaust consumers (Allenby and Rossi, 1989); as such, profiles must be limited in order to eliminate choice fatigue in participants. At the same time, based on the principle of random design, the attributes and attribute levels of traceable pork were randomly combined to ensure balanced attribute level distribution while reducing choice fatigue. The Choice Experiment was designed as follows: Firstly, the 15 main effects of the four traceable pork attributes shown in Table 1 to be studied were identified. In addition, 16 two-way interactions could be obtained between the different levels of each information attribute, which, together with the above-mentioned 15 effects, require a total of 31 degrees of freedoms. Secondly, 10 different versions of questionnaires were developed, with 10 tasks in each version. Each task contained two traceable pork profiles and a no-choice option. Thus, participants needed to compare 20 traceable pork profiles, which did not exceed the maximum quantity of profiles to avoid choice fatigue and satisfy minimum requirements for degrees of freedom. The final Choice Experiment design is shown in Figure 3. At the end of the experiment, each participant was asked demographic questions, such as gender, age, marital status, and educational background, as well as about weekly household consumption, to investigate the possible impact of such factors on consumption preferences.

### Organization and Implementation

This experiment was conducted by trained postgraduates from a well-known local university through direct one-on-one interviews with participants. To ensure the randomness of the respondents, every third person coming into view was selected as the respondent (Wu et al., 2015b). It should be noted that if consumer surveys were to be carried out in a city where traceable food pilot projects have not been implemented, the investigator would need to explain relevant concepts in detail as consumers may be unfamiliar with the concept of traceable food. This would not only increase the time cost of investigation, but also the dependence of the survey results on the concept explanation of the investigator, which may lead to biased research findings. Here, the survey was carried out in Wuxi, Jiangsu. Wuxi is one of the earliest pilot cities for a traceable pork system, and thus residents have a certain understanding of traceable pork. Moreover, its per capita GDP reached 174 600 yuan in 2018, one of the highest in China and indicating a high level of economic development. The experiment was carried out in all five administrative regions of Wuxi, including Liangxi, Binhu, Huishan, Xishan, and Xinwu, with 70 participants aged 18~65 recruited face-to-face in a large supermarket^5^ in each administrative region. The entire experiment was conducted during 18–21 October 2018, resulting in 328 valid questionnaires.

### Demographics of Participants and Their Understanding of Animal Welfare in Pig Production

Table 2 shows the demographics of the 328 participants recruited in the study. Among all participants, women accounted for 52.74% of total samples, which accords with the actual situation in China, i.e., more women are responsible for food purchases in Chinese families. In total, 74.39% of participants were aged between 18 and 35 years, 56.10% were unmarried, and 54.27% had a bachelor's degree or above; 66.77 and 93.90% of participants had no children under 18 years old and no pregnant or breast-feeding women in their families, respectively; 85.06% of participants had a good or very good health condition; 53.96% of participants had an annual household income of more than 100 000 yuan; and 43.90, 24.09, and 21.95% of participants had three, four, and five or above family members, respectively. In addition, 64.94% of participants had a household pork consumption of 500–1 500 g per week. However, it should be noted that as the sample was limited to consumers in Wuxi, it may not be representative of all cities in China.

Table 3 shows the perceptions of the 328 participants regarding animal welfare in pork production. In general, participants were basically satisfied with pork quality and safety in the current market. Specifically, 66.16% of participants expressed no knowledge of animal welfare. However, 43.60, 51.52, and 77.13% of participants considered “ready access to a satisfactory diet”, “living in a well-ventilated pigsty that allows comfortable rest and activity”, and “access to immediate treatment when sick” to be very important, respectively. Moreover, 53.35 and 77.74% of participants believed that it was completely necessary to safeguard pig welfare and that safeguarding pig welfare helped improve pork quality, respectively.

**Table 3:** Participants' perceptions of animal welfare in pork production.

**Item**	**Category**	**Sample size (person)**	**Proportion**
Satisfaction with current pork quality and safety (1 = total dissatisfaction, 10 = total satisfaction)	1	7	2.13
	2	7	2.13
	3	24	7.32
	4	16	4.88
	5	69	21.04
	6	63	19.21
	7	67	20.43
	8	50	15.24
	9	12	3.66
	10	13	3.96
Knowledge of animal (e.g., pig) welfare	No knowledge	217	66.16
	Low knowledge	76	23.17
	Medium knowledge	20	6.10
	High knowledge	13	3.96
	Very high knowledge	2	0.61
Ready access to a satisfactory diet	Very unimportant	4	1.22
	Unimportant	2	0.61
	Neither important nor unimportant	45	13.72
	More important	134	40.85
	Very important	143	43.60
Living in a well-ventilated pigsty that allows comfortable rest and activity	Very unimportant	4	1.22
	Unimportant	2	0.61
	Neither important nor unimportant	47	14.33
	More important	106	32.32
	Very important	169	51.52
Access to immediate treatment when sick	Very unimportant	2	0.61
	Unimportant	1	0.31
	Neither important nor unimportant	21	6.40
	More important	51	15.55
	Very important	253	77.13
Whether or not it is necessary to safeguard the welfare of pigs and other animals	Completely unnecessary	5	1.53
	Unnecessary	12	3.66
	Uncertain	17	5.18
	Slightly necessary	119	36.28
	Completely necessary	175	53.35
Whether or not it helps improve pork quality and safety by safeguarding pig welfare	Unhelpful	6	1.83
	Slightly helpful	37	11.28
	Uncertain	30	9.15
	Helpful	147	44.82
	Very helpful	108	32.92

## Model Estimation

### Model Building

According to the Independence of Irrelevant Alternatives (IIA) hypothesis proposed by Luce (1959), *U*_*imt*_ is the utility obtained by participant *i* in situation *t* by selecting the *m*_*th*_ traceable pork profile from subset *J* of choice space *C*, and includes the deterministic term *V*_*imt*_ and stochastic term ε_*imt*_, that is:

(1) $$\begin{array}{r} U_{imt}=V_{imt}+\varepsilon_{imt} \end{array}$$

(2) $$\begin{array}{r} V_{imt}=\beta_{i}^{\prime }X_{imt} \end{array}$$

where β_*i*_ is the marginal utility vector of participant *i*, and *X*_*imt*_ is the attribute vector of the *m*_*th*_ traceable pork profile. The *m*_*th*_ traceable pork profile is chosen when *U*_*imt*_ > *U*_*int*_ is true for any *n* ≠ *m*. Therefore, the probability that participant *i* selects the *m*_*th*_ traceable pork profile in situation *t* can be expressed as follows:

(3) $$\begin{array}{r} P_{imt}=prob\left(V_{imt}+\varepsilon_{imt}>V_{int}+\varepsilon_{int};\forall n\in C,n\neq m\right)\\ \;=prob\left(\varepsilon_{int}<\varepsilon_{imt}+V_{imt}-V_{int};\forall n\in C,n\neq m\right) \end{array}$$

If it is assumed that ε_*imt*_ follows a type I extreme value distribution, then the model determined by (1) and (2) is known as the conditional logit model, so that the conditional probability in (3) can be converted into the following form (Train, 2003):

(4) $$\begin{array}{r} P_{imt}=\frac{\exp\left(\beta_{i}^{\prime }X_{imt}\right)}{\sum_{n}\exp\left(\beta_{i}^{\prime }X_{int}\right)} \end{array}$$

In theory, each participant knows their own β_*i*_ and ε_*imt*_, but they cannot be directly observed, so researchers can only give the unconditional probability by observing *X*_*imt*_ as follows:

(5) $$\begin{array}{r} P_{imt}=\int \frac{\exp\left(\beta^{\prime }X_{imt}\right)}{\sum_{n}\exp\left(\beta^{\prime }X_{int}\right)}f\left(\beta \right)d\left(\beta \right) \end{array}$$

where *f (*β*)* is the probability density of β. Equation (5) is the general form of the logit model, which is known as the random parameters logit or mixed logit model. If it is assumed that participant preferences for traceable pork are homogeneous, that is, when β_*i*_ = *b, f (*β*)* = 1; and when β_*i*_ ≠ *b*, *f (*β*)* = 0, then Equation (5) can be converted into a fixed parameters logit model. As heterogeneous consumer preferences for traceable pork are more in line with reality, and the fixed parameter logit model may not meet the IIA assumption, the random parameters logit model is commonly used to assess consumer preferences in the field of food safety.

### Model Estimation

Based on the Choice Experiment, *Y*_*i*_ is the vector of the traceable pork profile chosen by participant *i* in different situations, that is, $Y_{i}^{\prime }=\left(Y_{i1},Y_{i2},\cdots \;,Y_{iT}\right)$. In this study, participants had a total of 10 tasks, representing their choices in 10 periods. Assuming that participants' preferences will not change in a short period of time, the conditional probability of selecting *Y*_*i*_ is as follows:

(6) $$\begin{array}{r} P\left(Y_{i}|X_{i},\beta_{i}\right)=\overset{10}{\underset{t=1}{\prod }}\frac{\exp\left(\beta_{i}^{\prime }X_{iY_{it}t}\right)}{\overset{4}{\underset{j=1}{\sum }}\exp\left(\beta_{i}^{\prime }X_{ijt}\right)} \end{array}$$

The unconditional probability is the integral of all β values in Equation (6), and can be specified as follows:

(7) $$\begin{array}{r} P\left(Y_{i}|X_{i}\right)=\int P\left(Y_{i}|X_{i},\beta \right)f\left(\beta \right)d\beta \end{array}$$

As Equation (7) is non-linear, maximum-likelihood estimation is a common estimation method. However, maximum-likelihood estimation can only be used to estimate the fixed parameters logit model, and whether its iteration is convergent is closely related to the setting of the initial value. More importantly, it is difficult to determine whether the result is globally or locally optimal (Train, 2003). Bayesian inference can be used to estimate group preferences. It provides better consistency and validity than maximum-likelihood estimation and does not require the calculation of an optimal solution, so that the related maximum-likelihood estimation defects can be avoided (Train, 2003). Therefore, hierarchical Bayesian inference was adopted in this paper.

If β_*i*_ is the score vector of participant *i*, which is in line with random effects distribution, then expectation is the function of covariant ω_*i*_, that is:

(8) $$\begin{array}{r} \beta_{i}=\Gamma^{\prime }\omega_{i}+\varepsilon_{i}\\ \varepsilon_{i}\sim \;MVN\left(0,V_{\beta }\right) \end{array}$$

where Γ is the regression coefficient matrix. If the covariant is not considered, namely, to make Γ = 0, then β_*i*_ ~ *MVN*(0, *V*_β_). In this paper, we assumed that *V*_β_ follows the inverse Wishart distribution, that is:

(9) $$\begin{array}{r} {\left(V_{\beta }\right)}^{-1}\sim \;W\left(v_{0},V_{0}\right) \end{array}$$

Based on the Bayesian rule, the posterior distribution of β_*i*_ is expressed as:

(10) $$\begin{array}{r} h\left(\beta_{i}|Y_{i},X_{i},\bar{\beta },V_{\beta }\right)\infty P\left(Y_{i}|X_{i},\beta_{i}\right)\pi \left(\beta_{i}\right) \end{array}$$

where π(β_*i*_) is the prior distribution of β_*i*_.

### Iterative Process

Hierarchical Bayesian inference can be expressed in hierarchical form as:

(11) $$\begin{array}{r} Y|X,\beta \end{array}$$

(12) $$\begin{array}{r} \beta |\omega ,\Gamma ,V_{\beta } \end{array}$$

(13) $$\begin{array}{r} \Gamma |a,A \end{array}$$

(14) $$\begin{array}{r} V_{\beta }|v_{0},V_{0} \end{array}$$

where Equations (13) and (14) are the hyper-parameters of prior distribution. The iterative Markov chain process in Equations (11)–(14) is as follows: (1) For each participant, extract β after obtaining *Y* and *X*, and then repeat for all participants; (2) extract Γ after obtaining β and *V*_β_ at the individual level; (3) extract *V*_β_ after obtaining β and Γ; (4) repeat the above process.

### Model Results and Analysis

Table 4 shows the assignment of the main variables. Here, we used effect coding for the assignment of the level variables of the four attributes of traceable pork, i.e., traceability information, animal welfare, place of origin, and price. We also assumed that the coefficients of the no-choice option, interaction terms, and price were fixed, and the parameters of other attributes were stochastic and normally distributed (Ubilava and Foster, 2009). The parameter estimation results of the model are shown in Table 5.

**Table 4:** Variable assignment.

**Variable**	**Variable assignment**
HITRACE	HITRACE = 1; METRACE = 0; LOTRACE = 0
METRACE	HITRACE = 0; METRACE = 1; LOTRACE = 0
LOTRACE	HITRACE = 0; METRACE = 0; LOTRACE = 1
NOTRACE	HITRACE = −1; METRACE = −1; LOTRACE = −1
PHYSICAL	PHYSICAL = 1; ENVIR = 0; HEALTH = 0
ENVIR	PHYSICAL = 0; ENVIR = 1; HEALTH = 0
HEALTH	PHYSICAL = 0; ENVIR = 0; HEALTH = 1
NOWELFARE	PHYSICAL = −1; ENVIR = −1; HEALTH = −1
LOCORIGIN	LOCORIGIN = 1; OTHORIGIN = 0
OTHORIGIN	LOCORIGIN = 0; OTHORIGIN = 1
NOORIGIN	LOCORIGIN = −1; OTHORIGIN = −1
PRICE1	PRICE1 = 1; PRICE2 = 0; PRICE3 = 0; PRICE4 = 0
PRICE2	PRICE1 = 0; PRICE2 = 1; PRICE3 = 0; PRICE4 = 0
PRICE3	PRICE1 = 0; PRICE2 = 0; PRICE3 = 1; PRICE4 = 0
PRICE4	PRICE1 = 0; PRICE2 = 0; PRICE3 = 0; PRICE4 = 1

**Table 5:** Hierarchical Bayesian iteration results.

**Main effect and interaction**	**Estimated coefficient**	**Standard error**
PRICE1	0.1663	0.4437
PRICE2	0.3177	0.4412
PRICE3	0.2595	0.4486
PRICE4	−0.3518	0.4536
HITRACE	1.0323**	0.0821
METRACE	0.6193**	0.0718
LOTRACE	−0.0002	0.0662
PHYSICAL	0.4429**	0.0709
ENVIR	0.2496**	0.0656
HEALTH	0.7801**	0.0780
LOCORIGIN	0.7888**	0.0697
OTHORIGIN	0.3943**	0.0654
HITRACE × PHYSICAL	0.3549**	0.1165
HITRACE × ENVIR	−0.1069	0.1180
HITRACE × HEALTH	−0.1878	0.1343
HITRACE × LOCORIGIN	0.1812	0.1021
HITRACE × OTHORIGIN	−0.0378	0.1018
METRACE × PHYSICAL	−0.0917	0.1210
METRACE × ENVIR	0.0650	0.1170
METRACE × HEALTH	−0.0716	0.1349
METRACE × LOCORIGIN	0.0097	0.1022
METRACE × OTHORIGIN	−0.0538	0.1005
LOTRACE × PHYSICAL	−0.0076	0.1199
LOTRACE × ENVIR	0.1981	0.1268
LOTRACE × HEALTH	−0.1115	0.1425
LOTRACE × LOCORIGIN	−0.1228	0.1015
LOTRACE × OTHORIGIN	−0.0995	0.1028
PHYSICAL × LOCORIGIN	0.0300	0.1092
PHYSICAL × OTHORIGIN	−0.0011	0.0994
ENVIR × LOCORIGIN	0.0573	0.1056
ENVIR × OTHORIGIN	−0.3082**	0.1002
HEALTH × LOCORIGIN	−0.2313*	0.1179
HEALTH × OTHORIGIN	0.3230**	0.1084
No-Choice Option	−1.6463**	0.4448
log likelihood	−2 763.1703	
AIC	5 618.3	

** and * represent coefficients significant at 1 and 5% levels, respectively.

As seen in Table 5, the regression results showed that the no-choice option was significant at the 1% level and the estimated coefficient was negative. In addition, the estimated coefficients of HITRACE, METRACE, PHYSICAL, ENVIR, HEALTH, LOCORIGIN, and OTHORIGIN were significant at the 1% level and were positive. As also shown in Table 5, HITRACE had the highest marginal utility (1.0323) among all traceable pork attributes, followed by LOCORIGIN (0.7888) and HEALTH (0.7801), which showed similar marginal utility. In addition, the order for marginal utility of other attribute levels was METRACE, PHYSICAL, OTHORIGIN, and ENVIR, i.e., 0.6193, 0.4429, 0.3943, and 0.2496, respectively. Therefore, the order of consumer preferences for traceable pork attributes was HITRACE, LOCORIGIN, HEALTH, METRACE, PHYSICAL, OTHORIGIN, and ENVIR.

The regression results of the price variable in Table 5 show that the estimated coefficients of all four price levels were positive but non-significant. This may be because the set price attributes had a relatively small effect on the marginal utility of traceable pork consumption compared with traceability information, animal welfare, and place of origin in the context of the four attributes and attribute levels set in the paper. Moreover, the marginal utilities of the four price levels reveal that the coefficients of the price attribute were not monotone. The marginal utility of the price attribute was not highest when the price was lowest (*PRICE1* = 14 yuan/500 g), but was highest at 15.4 yuan/500 g. However, when the price was higher than 15.4 yuan/500 g, marginal utility decreased with the increase in price, consistent with the Law of Demand in classical economics, that is, consumer demand decreases with the increase in commodity prices. The marginal utility not being highest when the price of traceable pork was lowest is probably because participants paid more attention to pork quality and safety. Better pork quality and safety can be safeguarded by a higher level of traceability information, which means a higher price. Comparatively speaking, it is difficult to safeguard the quality and safety of traceable pork at the lowest price, and thus its marginal utility was lower than that at 15.4 yuan/500 g. By further calculating the difference between the maximum and minimum marginal utility of each attribute of traceable pork divided by the sum of the corresponding differences of all attributes, we obtained the relative importance of each traceable pork attribute (Xu et al., 2019): i.e., traceability information, 39.30%; price, 25.49%; animal welfare, 20.19%; and place of origin, 15.02%.

In terms of interactions, the interactions HITRACE × PHYSICAL and HEALTH × OTHORIGIN were significant at the level of 1%, with positive coefficients. Thus, a complementary relationship existed between HITRACE and PHYSICAL and between HEALTH and OTHORIGIN. When the label only shows dietary welfare of pigs, that is, information on food and drinking water in the farming process, the addition of high-level traceability information that covers farming, slaughter and processing, and distribution and sales as a supplement can reduce consumer concerns about the risk of pork. Thus, there is a complementary relationship between HITRACE and PHYSICAL. When the label shows that traceable pork is non-indigenous, pigs cannot be guaranteed treatment when they are sick in the farming process, so the health welfare label is also required to ensure the safety of such pork. Thus, there is also a complementary relationship between HEALTH and OTHORIGIN. In contrast, the HEALTH × LOCORIGIN interaction was significant at the 5% level, with a negative coefficient. This suggests that the indigenous label guarantees access to medical treatment, which is the information contained in the health welfare label, resulting in a strong relationship between HEALTH and LOCORIGIN. Lastly, the ENVIR × OTHORIGIN interaction was significant at the 1% level, with a negative coefficient, indicating that a substitutional relationship exists between ENVIR and OTHORIGIN. The ex ante risk assessment carried out by participants on traceable pork based on the non-indigenous label already covered information about the habitat environment of pigs in the farming process, so OTHORIGIN can substitute ENVIR.

## Main Conclusions and Prospects

In this study, consumer preferences for traceable pork attributes, including traceability information, animal welfare, place of origin, and price, at different levels, were examined in 328 consumers in downtown Wuxi, Jiangsu Province, China, using a Choice Experiment and Bayesian inference. The main conclusions are as follows:

Firstly, when asked directly, 77.74% of participants claimed that safeguarding pig welfare was helpful or very helpful for improving pork quality and safety. The Choice Experiment results also showed that although the marginal utility of health welfare was lower than that of high-level traceability information, it was similar to that of place of origin, and higher than that of middle-level traceability information and other attribute levels. Consumer preference for health welfare was lower than that for traceability information. This may be due to their perceptions of higher traceability information than of health welfare as perception determines behavior. The calculation results showed that the relative importance of animal welfare to consumers was higher than that of place of origin among traceable pork attributes with the function of *ex ante* quality assurance. Therefore, including the animal welfare attribute in the form of a label may better meet consumer demand for pork safety and quality information than including the place of origin attribute. This conclusion accords with that of Yuta et al. (2018), i.e., most consumers prefer to purchase animal-derived food with an animal welfare label. This is because the animal welfare label provides more information than the place of origin label. For example, the health welfare attribute reflects information on disease treatment in the farming process of pigs, which is more helpful for consumers to determine pork quality and safety.

Secondly, we found a complementary relationship between high-level traceability information and dietary welfare. This finding suggests that the risk of pork with an animal welfare label only is still uncontrollable, and traceability information that covers farming, slaughter and processing, and distribution and sales should be included as a supplement. Therefore, to ensure the function of the animal welfare label, a label system that combines the traceability information attribute, which enables ex post traceability, and the animal welfare attribute, which enables ex ante quality assurance, may better meet market demand. This conclusion is consistent with that of Wu et al. (2015b). This is because complete traceability information covering farming, slaughter and processing, and distribution and sales ensures that sub-standard food can be effectively recalled, thereby safeguarding pork safety and quality. Thirdly, although the importance of the animal welfare information attribute was lower than that of the price attribute, the marginal utilities of health welfare and dietary welfare were higher than those of all price levels. This indicates that consumers accept a higher price as a result of increased production costs due to the inclusion of animal welfare information. This is mainly because consumers are highly concerned about food safety. This conclusion accords with that of previous studies in other countries. For example, Yuta et al. (2018) reported that nearly 90% of Japanese consumers are willing to pay a certain premium for beef with an animal welfare label. Spain et al. (2018) found that American consumers are willing to pay a premium of 0.79 US dollars (32%) for eggs with animal welfare information. Thus, consumer demand already exists for the setting of an animal welfare attribute for traceable animal-derived food in the context that consumers are highly concerned about the safety of animal-derived food in China. Due to the COVID-19 pandemic, China has recently approved the integration of an animal welfare attribute into traceability market systems of new animal-derived foods. The government should encourage manufacturers to produce diverse traceable animal-derived food not only to protect animal welfare and promote the construction of an ecological civilization, but also to develop new animal-derived food markets to satisfy different levels of consumer demand.

There are some study limitations to mention. The current experiment was a hypothetical experiment, which did not include actual payment by consumers. Given the characteristics of stated preference, consumers tend to exaggerate or falsely express their consumption behavior, which may differ from their behavior under a real market environment. Therefore, non-hypothetical experiments should be used in future studies. In addition, as the sample was limited to consumers in Wuxi, Jiangsu Province, further research with a wider scope is required to confirm the universality of our findings.

## Data Availability Statement

The original contributions presented in the study are included in the article/supplementary material, further inquiries can be directed to the corresponding author.

## Ethics Statement

The studies involving human participants were reviewed and approved by Jiangnan University. The patients/participants provided their written informed consent to participate in this study.

## Author Contributions

LW: conceptualization and validation. EH: data curation and writing—review and editing. LK: formal analysis. MC: writing—original draft.

## Conflict of Interest

The authors declare that the research was conducted in the absence of any commercial or financial relationships that could be construed as a potential conflict of interest.

## Footnotes

^1^Protection of Food Safety! Top Ten Food Crime Cases in the First Half Uncovered by Zhejiang Provincial Public Security Department, source: China.com.cn (http://zjnews.china.com.cn/yuanchuan/2019-07-03/178907.html).

^2^In China, many food safety and quality incidents are caused by dishonest acts of food producers and operators, e.g., food fraud and adulteration. Therefore, the integrity of food producers and operators in a region directly affects consumer expectations of food safety in that region. The term “place of origin” here not only indicates a geological range, but also covers consumer assessment of the integrity of food producers and operators in that region.

^3^“Place of origin” included in the traceable information system in this study refers only to the location of pig farming.

^4^The investigator prepared three samples of pure lean pork with “fresh, moderate, and unattractive” appearance by adding food coloring and showed them to the participants during the experiment for comparison.

^5^The selected supermarkets were Carrefour in Liangxi, Auchan in Binhu, Tesco in Huishan, RT-Mart in Xishan, and Hualian in Xinwu. All have a business area larger than 3 000 m^2^ and average annual flow of 1.5 to 2 customers/m^2^.

## References

[B1] Abidoye B. O. Bulut H. Lawrence J. D. Mennecke B. Townsend A. M. (2011). U.S. consumers' valuation of quality attributes in beef products. J. Agric. Appl. Econ. 43, 36–45. 10.1017/S1074070800004016

[B2] Alfnes F. Chen X. Rickertsen K. (2018). Labeling farmed seafood: a review. Aquaculture Econ. Manage. 22, 1–26. 10.1080/13657305.2017.1356398

[B3] Allenby G. M. Rossi P. E. (1989). Marketing models of consumer heterogeneity. J. Econ. 89, 57–78. 10.1016/S0304-4076(98)00055-4

[B4] Bai J. Zhang C. Jiang J. (2013). The role of certificate issuer on consumers' willingness-to-pay for milk traceability in China. Agric. Econ. 44, 537–544. 10.1111/agec.12037

[B5] Beltrán-Alcrudo D. Arias M. Gallardo C. Kramer S. Penrith M. L. (2017). African swine fever: detection and diagnosis—A manual for veterinarians. Rome: FAO Animal Production and Health Manual No.19, Food and Agriculture Organization of the United Nations (FAO). 88.

[B6] Chang K. L. Xu P. Underwood K. Mayen C. Langelett G. (2013). Consumers' willingness to pay for locally produced ground beef: a case study of the rural Northern Great Plains. J. Int. Food Agribus. Mark. 25, 42–67. 10.1080/08974438.2013.724002

[B7] European Food Safety Authority (ESFA) (2019). Animal Welfare: Introduction. Available online at: https://www.efsa.europa.eu/en/topics/topic/animal-welfare last (accessed May 15, 2019)

[B8] Farm Animal Welfare Council: Five Freedoms, FAWC (2009). Available online at: http://www.fawc.org.uk/freedoms.htm (accessed July 20, 2019)

[B9] Gavinelli A. Rhein C. Ferrara M. (2007). European policies on animal welfare and their effects on global trade. Farm Policy J. 4, 11–21.

[B10] Golan E. H. Crissoff B. Kuchler F.. (2003). Traceability in the US food supply: dead end or superhighway? Choice 18, 47–64. Available online at: https://www.researchgate.net/publication/227364624

[B11] Grace D. Jones B. McKeever D. Pfeiffer D. (2011). Zoonoses (Project 1): Wildlife/Domestic Livestock Interactions. ILRI.

[B12] Grace D. Mutua F. Ocungo P. Kruska R. Ogutu F. (2012). Mapping of Poverty & Likely Zoonoses Hotspots. Available online at: https://xueshu.baidu.com/usercenter/paper/show?paperid=8483e7c6d31381ccda24bbf16fff7637

[B13] Hobbs J. E. (2004). Information asymmetry and the role of traceability systems. Agribusiness 20, 397–415. 10.1002/agr.20020

[B14] Hou B. Wu L. H. Chen X. Zhu D. Ying R. Tsai F. S. (2019). Consumers' willingness to pay for foods with traceability information: ex-ante quality assurance or ex-post traceability? Sustainability 11, 1464–1478. 10.3390/su11051464

[B15] Iannetti L. Neri D. Santarelli G. A. Cotturone G. Vulpiani M. P. Salini R.. (2019). Animal welfare and microbiological safety of poultry meat: impact of different at-farm animal welfare levels on at-slaughterhouse Campylobacter and Salmonella contamination. Food Control 9, 109–116. 10.1016/j.foodcont.2019.106921

[B16] Kher S. V. Jonge J. D. Wentholt M. T. A. Deliza R. de Andrade J. C. Cnossen H. J.. (2013). Consumer perceptions of risks of chemical and microbiological contaminants associated with food chains: a cross-national study. Int. J. Consum. Stud. 37, 73–83. 10.1111/j.1470-6431.2011.01054.x

[B17] Lai J. Wang H. H. Ortega D. L. Olynk Widmar N. J. (2018). Factoring Chinese consumers' risk perceptions into their willingness to pay for pork safety, environmental stewardship, and animal welfare. Food Control 85, 423–431. 10.1016/j.foodcont.2017.09.032

[B18] Lancaster K. J. (1996). A new approach to consumer theory. J. Polit. Econ. 4, 132–157. 10.1086/259131

[B19] Lemos Teixeira D. Larraín R. Hötzel M. (2018). Are views towards egg farming associated with Brazilian and Chilean egg consumers' purchasing habits? PLoS ONE. 13:e0203867. 10.1371/journal.pone.0203867 30265672 PMC6161848

[B20] Li J. Chai M. Yuan S. Zhao M. Cui P. (2019). Prevalence and control of zoonosis. Anim. Husb Vet. Med. Today 35:34.

[B21] Lim K. H. Hu W. Maynard L. J. Goddard E. (2014). A taste for safer beef? how much does consumers' perceived risk influence willingness to pay for country-of-origin labeled beef. Agribusiness 30, 17–30. 10.1002/agr.21365

[B22] Loebnitz N. Loose S. M. (2015). Impacts of situational factors on process attribute uses for food purchases. Food Qual. Preference 44, 84–91. 10.1016/j.foodqual.2015.03.014

[B23] Loureiro M. L. Umberger W. J. (2007). A choice experiment model for beef: what US consumer responses tell us about relative preferences for food safety, country-of-origin labeling and traceability. Food Policy 32, 496–514. 10.1016/j.foodpol.2006.11.006

[B24] Louviere J. J. Flynn T. Carson R. (2010). Discrete choice experiments are not conjoint analysis. J. Choice Model. 3, 57–72. 10.1016/S1755-5345(13)70014-9

[B25] Louviere J. J. Hensher D. A. Swait J. D. (2000). Stated choice methods: analysis and applications. Cambridge, UK: Cambridge University Press. 10.1017/CBO9780511753831

[B26] Luce R. D. (1959). Individual Choice Behavior: A Theoretical Analysis. New York: John Wiley&Sons.

[B27] Markova-Nenova N. Wätzold F. (2018). Fair to the cow or fair to the farmer? The preferences of conventional milk buyers for ethical attributes of milk. Land Use Policy 79, 223–239. 10.1016/j.landusepol.2018.07.045

[B28] Merlino V. M. Borra D. Girgenti V. Dal Vecchio A. Massaglia S. (2018). Beef meat preferences of consumers from Northwest Italy: analysis of choice attributes. Meat Sci. 143, 119–128. 10.1016/j.meatsci.2018.04.023 29738962

[B29] Nguyen H.D. My, Demontc M. Van Looa E. J. de Guia A. Rutsaert P. Tuan T. H.. (2018). What is the value of sustainably-produced rice? consumer evidence from experimental auctions in Vietnam. Food Policy 79, 283–296. 10.1016/j.foodpol.2018.08.004

[B30] Office International Des Epizooties (OIE) (2015). Animal Welfare. Available online at: http://www.oie.int/en/animal-welfare/ (accessed August 19, 2019).

[B31] Opara (2003). Traceability in agriculture and food supply chain:a review of basic concepts, technological implications, and future prospects. J. Food, Agric. Environ. 1, 101–106.

[B32] Reardon T. Timmer C. P. (2012). The economics of the food system revolution. Annu. Rev. Resour. Econ. 4, 225–264. 10.1146/annurev.resource.050708.144147

[B33] Regattieri A. Gamberi M. Manzini R. (2007). Traceability of food products: general framework and experimental evidence. J. Food Eng. 81, 347–356. 10.1016/j.jfoodeng.2006.10.032

[B34] Ren D. (2006). Interpretation of Animal Husbandry Law of the People's Republic of China. Beijing: China Legal Publishing House.

[B35] Spain C. V. Freund D. Mohan-Gibbons H. Meadow R. G. Beacham L. (2018). Are they buying it? United states consumers' changing attitudes toward more humanely raised meat, eggs, and dairy. Animals 8, 1–14. 10.3390/ani8080128 30044402 PMC6116027

[B36] Train K. (2003). Discrete Choice Methods With Simulation. New York: Cambridge University Press. 10.1017/CBO9780511753930

[B37] Ubilava D. Foster K. (2009). Quality certification vs. product traceability: consumer preferences for informational attributes of pork in Georgia. Food Policy 34, 305–310. 10.1016/j.foodpol.2009.02.002

[B38] Wang C. W. Gu H. Y. (2016). Animal welfare cognition and food safety. J. Financ. Econ. 42, 21–30. 10.16538/j.cnki.jfe.2016.12.002

[B39] Wang H. M. Ni C. J. Xu R. Z. (2011). An empirical study on the consumers' willingness to pay for food quality and safety labels: a case study of pork consumption in Nanjing. J. Nanjing Agric. Univ. (Soc. Sci.). 11. 10.3969/j.issn.1671-7465.2011.01.004

[B40] Wang J. Yue H. Zhou Z. (2017). An improved traceability system for food quality assurance and evaluation based on fuzzy classification and neural network. Food Control 79, 363–370. 10.1016/j.foodcont.2017.04.013

[B41] Weerd H. A. V. D. Day J. E. L. (2009). A review of environmental enrichment for pigs housed in intensive housing systems. Appl. Anim. Behav. Sci. 116, 1–20. 10.1016/j.applanim.2008.08.001

[B42] Wu L. Wang H. Zhu D. (2015b). Analysis of consumer demand for traceable pork in China based on a real choice experiment. China Agric. Econ. Rev. 7, 303–321. 10.1108/CAER-11-2013-0153

[B43] Wu L. Wang S. Zhu D. Hu W. Wang H. (2015c). Chinese consumers' preferences and willingness to pay for traceable food quality and safety attributes: the case of pork. China Econ. Rev. 35, 121–136. 10.1016/j.chieco.2015.07.001

[B44] Wu L. H. Gong X. R. Chen X. J. (2018). customer preferences for traceable information attributes with functions of ex ante quality assurance and ex post traceability. China Popul. Res. Environ. 28, 148–160.

[B45] Wu L. H. Gong X. R. Qin S. S. Chen X. J. Zhu D. Hu W.. (2017). Consumer preferences for pork attributes related to traceability, information certification, and origin labeling: based on China's Jiangsu province. Agribusiness 14, 1–19. 10.1002/agr.21509

[B46] Wu L. H. Wang H. S. Liu X. L. (2014). Consumers' willingness to pay for pork of combined traceable information levels. China Popul. Res. Environ. 24, 35–45.

[B47] Wu L. H. Xu L. L. Yin S. J. (2015a). China Development Report on Food Safety. Peking University Press.

[B48] Xu L. Yang X. Wu L. Chen X. Chen L. Tsai F. S. (2019). Consumers' willingness to pay for food with information on animal welfare, lean meat essence detection, and traceability. Int. J. Environ. Res. Public Health 16:3616. 10.3390/ijerph16193616 31561621 PMC6801471

[B49] Yin S. Li Y. Xu Y. Chen M. Wang Y. (2017). Consumer preference and willingness to pay for the traceability information attribute of infant milk formula: evidence from a choice experiment in China. Br. Food J. 119, 1276–1288. 10.1108/BFJ-11-2016-0555

[B50] Yuta S. Kazato O. Yosuke C. Hiroyuki H. (2018). How do human values influence the beef preferences of consumer segments regarding animal welfare and environmentally friendly production? Meat Sci. 11, 41–52. 10.1016/j.meatsci.2018.07.030 30103081

[B51] Zhong Y. Q. Wu L. H. (2018). A Study on Producer Behavior of Pig Farmers from the Perspective of Food Quality and Safety. People' s Publishing House: Beijing.

